# The Association between the Dental Status and Tongue Thrust Habits of Latvian Preschool Children and Their Mothers’ Oral Health Knowledge

**DOI:** 10.3390/diagnostics14060605

**Published:** 2024-03-12

**Authors:** Lilian Tzivian, Dace Priede, Valdis Folkmanis, Ieva Henkuzena

**Affiliations:** 1 Institute for Clinical and Prevention Medicine, University of Latvia, Raina blv. 19, 1586 Riga, Latvia; 2 Faculty of Medicine, University of Latvia, 1586 Riga, Latvia

**Keywords:** preschool children, child oral health, oral habits, medical communication

## Abstract

Objectives: The aim of this study was to describe the factors that affect the normal dental development of preschool children in Latvia, and to investigate sources that mothers use to get information on children’s oral health. Materials and Methods: A cross-sectional study was performed in two kindergartens in Latvia (cluster sampling). The study sample comprised 141 child–mother pairs of preschool children aged 4–7 years. The dental situation of all children was assessed including evaluation by an orthodontist and a speech therapist, and mothers of children filled out the survey on oral health-related habits and information about it. Statistical analysis: We described individually factors related to orthodontal situations, children’s speech problems, and factors that can affect tongue thrust. We investigated relationships between sources of mothers’ information and oral health-related behaviors using univariate (Kruskal–Wallis test, a chi-squared test, a Fisher test, or Cramer’s V test) and multivariate analyses. We built a multiple logistic regression model adjusted for the demographic and oral health-related factors to investigate the factors associated with tongue thrust. Results of multiple logistic regression were presented with odds ratio (OR) and 95% confidence intervals (CI). Results: In total, 36.9% of children grazed vegetables several times a week, and 61.0% cleaned their teeth twice a day. Of mothers, 12% did not receive any information about child dental care from their general physician, and 23.4% found the received information insufficient. A total of 43.3% of mothers received oral health-related information from friends, and it was significantly related to less carbonated water (*p* < 0.01), more help during teeth cleaning (*p* = 0.03), starting cleaning teeth in earlier age (*p* = 0.03), and more frequent visits to a child dentist (*p* = 0.03). Conclusions: A lack of knowledge was found to be prominent in mothers of kindergarten children in Latvia, and most of them received information not from official sources such as their general physician. This can be related to some problems in oral health behaviors and oral health-related diseases. Communication among dental health specialists, state authorities, and families is crucial for the improvement of children’s dental situation.

## 1. Introduction

Oral health is a multidimensional concept that implies the absence of pathological processes in oral hard and soft tissues [1]. Accordingly, it can be adversely affected by poor hygiene and diet, as well as some deleterious habits such as thumb or digit sucking [2], nail biting [3], operations or injuries in the head and neck areas [4], or mouth breathing [5].

The growing amount of information about dental care and the availability of new technologies that allow better dental treatment have resulted in considerable improvements in oral health. However, in some countries—including the Baltic states (Estonia, Lithuania, and Latvia)—this process is slower than in others. In Latvia, for instance, not enough is known about oral health and care, and the quality of the available information is inadequate. Moreover, in 2019, Poskevicios found that only 651 scientific articles related to both pediatric and adult dentistry were published between 1996 and 2018 [6]. Only 210 of these studies were conducted in Latvia, and only six authors had more than 10 publications [6]. This finding suggests that, in this country, research on this topic is insufficient, and the communication between the scientific community and the general public in the field of oral health is poor.

Regarding children’s (especially preschoolers’) oral health and its promotion in Latvia, the situation is even more problematic, as some inappropriate practices that date back to the USSR era are still followed, due to which children’s dentistry is relatively poor [7]. For example, one of the recommendations is not to clean children’s teeth before they reach 12 months of age, and generally deciduous teeth are not adequately cared for because they will be replaced eventually by permanent dentition. Unfortunately, these mistakes are still common in the Latvian society and parents are unaware of the importance of proper dental care in preschool children [8]. In addition, there is a lack of communication between parents and pediatric dentists even in cities where most of the Latvian population lives (68.6% according to the 2020 Worldometer data) [9].

We hypothesized that mothers in Latvia do not have enough information and knowledge concerning the dental health and care of their young children. For example, mothers are not aware of the necessity to brush baby teeth or to take a child of kindergarten age to the dentist. This inattention to the early development of the child’s teeth can lead to tongue changes or problems with speech development. To understand the extent of these issues, we conducted the present study to identify as many factors as possible that can affect normal dental development. Our second hypothesis was that mothers do not receive sufficient information on child dental care from their general physician. To address these issues, we investigated the sources of information that mothers use to fill this gap and sought to assess the effect that this practice has on the dental health-related behaviors of mothers and children. We investigated other sources of information mothers use to fill this gap and sought to assess the effect that this practice has on the dental health-related behaviors of mothers and children. In addition, we assumed a lack of awareness of mothers about oral health-related behaviors such as thumb sucking or cleaning teeth and children’s oral health behaviors leading to some further oral health problems and diseases. We aimed to evaluate the association between the major problem found in the children we investigated—an infantile tongue thrust—and oral care-related diseases and oral health behaviors in Latvian preschool children.

## 2. Materials and Methods

### 2.1. Study Population and Design

Our cross-sectional study was performed in two kindergartens, respectively located in the capital of Latvia, Riga, and in the economically stable region of Latvia—Ikshkile—located 27 km away from Riga. These regions were chosen based on their economic similarity, as we assumed that the socioeconomic status of parents can affect their knowledge of children’s oral health. The two kindergartens were subsequently chosen randomly as a cluster that represents children in a good socioeconomic situation and can thus adequately represent all children living in big Latvian cities. The oral condition of preschool children who met the study inclusion criteria (4–7 years old and present at the initial visit to the kindergarten, and whose mothers agreed to participation) was assessed during six visits. Children who exhibited any psychological or behavioral problems that precluded physical investigation were excluded, as were those whose mothers were unwilling to take part in the study. The data obtained via six subsequent examinations was supplemented by the demographic and oral health-related data provided by the mothers who filled out the questionnaire designed for this purpose. Data received during the investigation was registered using a computerized management tool, developed specifically for this study, and further data from the mothers’ survey was matched to the data of their children. The database on individual laptops and on the central computer was secured by an authorization module to protect the data from being accessed by unauthorized persons. The correctness of the data transmission was checked twice by two independent staff members. The study was approved by the Ethical Committee of the Institute of Clinical and Preventive Medicine, University of Latvia (approval No. 186/2017 from 18 December 2017).

### 2.2. Mothers’ Survey on Children’s Oral Health

As noted above, in parallel to the physical investigation of children, their mothers took part in a survey probing into the age and gender of their child, the family socioeconomic status, and the parents’ educational attainment. Additionally, mothers provided other information pertinent to potential orthodontal problems, including child delivery (natural or via Cesarean section), breastfeeding duration, and the age at which the child started attending kindergarten. Mothers also evaluated the child’s bad habits related to dental problems, such as nail biting, sleeping with an open mouth, bruxism during the night, thumb sucking, and vegetable grazing frequency. The survey also probed into the presence of some diseases during the child’s development such as prolonged colds, allergies, traumas, surgeries performed under full anesthesia, and otitis media. We further inquired about the sources of information that mothers use to learn about oral care and child feeding, as well as their level of satisfaction with the information received from the child’s physician. All these factors were important to describe the oral health situation of children in the two kindergartens, assuming that the chosen sample is representative of all Latvian children of this age.

### 2.3. Physical Investigation of the Children

Physical investigation of the children included evaluation by the children’s dentists and a speech therapist. The first was performed by two children’s dentists in purposely prepared working stations in the kindergarten, which were equipped with a dental lamp. The agreement between the children’s dentists was previously checked by performing a pilot study involving 10 children, whereby both physicians evaluated the same children after which the similarity of their evaluations was assessed using a reliability test. As sufficient agreement was reached, the remaining children were assessed by one of the children’s dentists only. The evaluation included a check for a tongue thrust during swallowing (infantile or normal), lip position (upper and lower lip individually: normal, hyper-tonus, short, and hypertrophy of *Frenulum labia sup*.), lip power (in grams), and breathing type (through the nose, mouth, or combined).

Evaluation by the speech therapist included assessment of speech defects, focusing on the child’s overall communication skills and ability to pronounce phonetical sounds, especially typical Latvian consonants *c*, *dzh*, *k*, *l*, *n*, *r*, *s*, *sh*, *sch*, *t*, and *v*, and the overall intelligibility of speech. For the purpose of statistical analyses, we grouped speech problems into the *non-normal* category and compared them with *normal* speech.

### 2.4. Statistical Analysis

Only the data pertaining to children that had completed all orthodontic status evaluations and whose mothers provided at least 90% of the required survey data were included in the statistical analysis. To fulfill the first aim of this study and to describe the oral health-related situation of Latvian preschool children, descriptive statistics were performed on all study variables. For lip power, we presented the median and range, as the distribution of this variable was not normal (Kolmogorov–Smirnov test, *p* < 0.01). For group variables, we calculated numbers and percentages for each group. We also described individual factors related to the orthodontal situation, children’s speech problems, and factors that can affect this situation (based on the mothers’ surveys). We investigated the relationships between each one of the five sources that mothers used for information on their child’s oral health (relatives, friends, general physician, internet, and books and magazines) and oral health-related behaviors using the chi-squared test.

To investigate the relationship between tongue thrust and other orthodontal and behavioral factors, we first performed a univariate analysis. For lip power, we used a Kruskal–Wallis test, a chi-squared test, a Fisher test, or Cramer’s V test for categorical variables. At this stage, we set the level of significance at α = 0.05. Next, a multiple logistic regression model was developed for tongue thrust and the variables that displayed the statistical significance in the univariate analysis. We considered the gender and age of the child as additional potentially important factors and incorporated them into all multiple logistic regression models, although they did not display statistical significance in the univariate stage. The final set of variables for the multiple logistic regression models included breathing, otitis media, age, and gender. We presented odds ratio (OR) and 95% confidence intervals (CI) for this stage of the analysis. All data was analyzed using Statistical Program for Social Studies (SPSS) software (version 26) [10].

## 3. Results

### 3.1. Study Population and Description of Factors Affecting Normal Dental Development

While the study initially involved 165 child–mother dyads, 24 dyads were eliminated prior to analyses due to more than 10% missing data. Thus, the final sample comprised 141 children (most of whom were boys, aged 3–4 years, who had been born naturally) and their mothers. Mothers were mostly highly educated, with high self-reported socioeconomic status. Children were mostly breast-fed for the first 12 months of life, and spoon-feeding was introduced at 6–9 months, while kindergarten attendance commenced at 1–3 years of age. Most of the children (85.8%) stopped being bottle-fed before the age of 1.5 years or had never been fed formulas. The majority of the children grazed vegetables several times a week, never grazed nails, slept with their mouths closed, and did not snore or suck a finger. Almost all children (98.5%) consumed carbonated drinks not more than several times a month and about 50% of the sample were given sweets not more than once a day.

Information related to oral hygiene was rather heterogeneous, as more than half of the studied children cleaned their teeth twice a day, but 7% would do it only if reminded by their parents. However, while none of the mothers selected “don’t clean teeth at all” as a response to this question, “clean each time after eating” was also never chosen. Half of the children cleaned their teeth by themselves, while parents checked the quality of the cleaning for the other half. Most parents were aware that children’s teeth should be cleaned as soon as the first tooth erupts. However, about 30% of parents indicated that they started cleaning their child’s teeth after their first birthday. More than half of parents (64.5%) took their child to a dentist within the 6-month period preceding the study, but almost 8% never planned such a visit (Table 1).

Physical investigations revealed that approximately half of the children (*n* = 75, 53.2%) had infantile tongue thrust during swallowing. Concerning diseases that can potentially harm dental health, 21.3% of children had allergies, 46.1% had long-lasting flu, 22% had otitis media, 17% had face/ head/ heck traumas, and 13.5% underwent surgery under general anesthesia prior to the current study. Most of the children had normal nose breathing (81.6%), and normal speech (62.4%), and did not have interdental speech (62.4%). Normal occlusion was observed in 35.5% of children. Lip power ranged from 100 to 700 g with a mean value of 346.9 g (standard deviation, SD, 133.2 g; median 300 g).

### 3.2. Sources of Information about Children’s Oral Health

In this economically stable region of Latvia, about 12% of mothers did not receive sufficient information about child dental care from their general physician or nurse. Moreover, only 27% of those who received such information found it useful. In addition, 37% of mothers did not indicate general physicians as their main source of information on child dental care, as most mothers relied on books or journals, followed by the internet, whereby about half of the mothers discussed child dental care with their friends (Table 1).

While mothers used all five sources (relatives, friends, general physician, internet, and books and magazines) to obtain knowledge on child oral health, only information provided by friends was significantly related to better oral health-related behaviors. Moreover, if mothers received such information from friends, their children drank significantly less carbonated water, received more help during teeth cleaning, started cleaning their teeth at an earlier age, and visited a dentist more frequently. These children were also less likely to suck their thumbs after the age of 1 year (*p =* 0.07) (Table 2). All other sources of information did not affect oral health-related behaviors.

### 3.3. Association between Tongue Thrust and Dental Care-Related Factors

In the univariate analysis, none of the sociodemographic variables was statistically significantly related to tongue thrust. Nevertheless, we included the child’s age (*p =* 0.23) and gender (*p* = 0.45) in the multiple regression models as these were deemed clinically important variables. Concerning diseases related to oral health, only otitis media exhibited a statistically significant relationship with tongue thrust (*p* = 0.02). In addition, breathing (*p* < 0.01), speech (*p* < 0.01), and interdental speech (*p <* 0.01) findings were significantly related to infantile tongue thrust (Table 3). We did not include the interdental speech in the multivariate analysis as these variables explain similar processes. None of the children with normal speech had lisping, and 32.1% of those with speech problems also had lisping (*p <* 0.01).

According to the fully adjusted multiple logistic regression model, mouth breathing, non-normal speech, and otitis media statistically significantly increased the likelihood of infantile tongue thrust (odds ratio, OR = 4.60 [confidence interval, CI 1.55; 13.65], OR = 3.43 [CI 1.58; 7.48], and OR = 2.60 [CI 1.04; 6.50], respectively). These factors explained 22.4% of the variance in changes in tongue thrust. Neither the child’s age nor gender was associated with tongue thrust (Table 4).

## 4. Discussion

In our study, we observed that, despite the insufficient knowledge that mothers of children in the two kindergartens where the data was gathered received from their general physicians, most of their children did not have problems with breathing, speech, or lisping. However, the inability to obtain adequate information from their physician likely contributed to some poor oral health outcomes reported by the mothers, such as drinking carbonated water, receiving more help during teeth cleaning, starting teeth cleaning at a younger age, and regularly visiting a pediatric dentist. We also noted that almost half of the children included in our study had infantile tongue thrust in swallowing, and this phenomenon was positively associated with speech, breathing, and otitis media.

In this study, we attempted to identify the main factors that can affect Latvian children’s oral health, as it is generally suboptimal due to some practices that have remained unchanged since the Soviet times. In addition, only a few studies on children’s oral health are performed each year in Baltic states, including Latvia [7,8,11]. Studies conducted on preschool children’s oral health in Baltic countries are particularly rare. For example, the most recent study performed in Lithuania dates back to 2014 [12]. It involved 503 preschool children as the authors aimed to identify factors that affect occlusion in young children. The last study in this field performed in Estonia dates back to 2005 [13], while Gudkina et al. performed a more recent study involving 38 and 39 Latvian children aged 6 and 12 years, respectively [14]. However, studies exploring the full panel of factors related to oral health have not, to our knowledge, been conducted to date. As a result, we can compare our findings with those presented by Korbmacher et al. in 2005 based on their research conducted in Germany, although children in this study were aged 1–19 years and the sample was not drawn from the general population, as all participants were referred to the Center for Manual Medicine because of dental problems [15]. In this study, 59.4% of children had a history of bad oral habits, while 71.0% had several orofacial problems, including open mouth at rest (55.7%), abnormal rest position of the tongue (71.9%), and infantile tongue thrust (61.6%). In our study sample, we observed infantile tongue thrust in 53.2% of children. While most of the children included in our analyses did not have bad oral health-related habits, we attribute this finding to the high socioeconomic status of mothers and their understanding of the importance of this practice. Similar to the results of our study, a study by Carli et al. (2023) observed a large number of orthodontic and oral health-related behavioral problems in Italian children [16]. Another aspect of our study—the relationship between infantile tongue thrust and breath and speech problems—was also completely in line with findings observed in other countries [17]. We see it as a positive side of our study to be able to confirm the results of studies performed previously in the population of children in Latvia. Finally, the information obtained on factors associated with tongue thrust allows us to make recommendations to public health authorities and to improve preschool children’s oral health.

Another aspect that adversely affects children’s oral health is the insufficient information that mothers receive from healthcare practitioners. However, this problem is not unique to the field of oral health, given that a lack of information on the possible harmful effects of soy-based formulas on child development has been previously reported [18,19]. Similarly, differing preferences regarding the communication of neonatal problems between parents and healthcare professionals can lead to suboptimal care [20]. These observations are in line with the findings yielded by our study, as most mothers stated that they did not receive any information from their general physicians, or it was insufficient, which led them to peruse other sources such as the internet or seek the help of their friends, which is also known from the literature [21]. However, given the clear benefits of a family-centered care approach, which requires the establishment of confidential relationships between caregivers and family members [22,23], this strategy should be adopted in dentistry as well in order to improve children’s oral health [24], health-related quality of life [25], and reduce the prevalence of tongue thrust.

As with any other field of medicine, dentistry is a fast-developing field of knowledge. The newest studies suggest that the use of artificial intelligence and the newest technologies can improve the management of orthodontic treatment and diagnostics [26,27,28], both in children and in adults. For example, they can help to assess the pressure of the tongue on teeth during swallowing—a measure that is nearly impossible without the newest technologies [29]. However, the inclusion of these technologies in diagnosis and treatment is not possible without proper evaluation of patient needs and information for patients on the procedures that are planned [30]. This makes communication between the caregiver and the patient extremely important. In the case of children’s oral care, the explanation of every process and procedure to the child’s mother becomes an essential part of the treatment and prevention processes. In our opinion, public health authorities and educators should pay specific attention to the communication issues in every medical field and prepare future dentists to provide explanations and dialogue with the patient. 

When interpreting the findings reported here, three important limitations that can affect the validity of the present study should be noted. First, we performed cluster sampling assuming the two kindergartens in which the study was performed were similar from the oral health perspective to other kindergartens located in cities and high-income areas of Latvia. Second, we adopted a cross-sectional design, as this allowed us to suggest reversed causality of the observed associations. Third, information on oral health-related behaviors was self-reported by mothers and could be prone to information (memory) bias.

The most important strength of this study is that it was the first attempt to investigate a large panel of factors related to children’s oral health in the Baltic region. Its further strength derives from the knowledge that the participating mothers gained, as they can use the information obtained as the basis for the oral health care they provide to their children in the future. Finally, the information obtained on factors associated with tongue thrust allows us to make recommendations to public health authorities and to improve preschool children’s oral health.

## 5. Conclusions

Despite the insufficient knowledge that Latvian mothers of young children received from their general physicians, most of the children attending the two kindergartens in which this study was conducted did not have problems with breathing, speech, or lisping. However, the lack of knowledge might be related to some poor oral health outcomes. Thus, to mitigate these negative outcomes, mothers should be better informed by state authorities about the latest guidelines on child oral health. Communication among dental health specialists, state authorities, and families is crucial for the improvement of children’s dental situation in the country.

## Figures and Tables

**Table 1:** Description of the study sample.

Variable, *N* = 141	Group	*N* (%)
Sociodemographic data and early-childhood conditions		
Child gender	Male	83 (58.9)
Child age	2–3 years 3–4 years 4–5 years	1 (0.7) 71 (50.4) 69 (48.9)
Mother’s education	Basic Secondary Professional Higher	2 (1.4) 31(22.0) 1 (0.7) 107 (75.9)
Socioeconomic status	Low Medium Good Very good	1 (0.7) 53 (37.6) 76 (53.9) 11 (7.8)
Way of giving birth	Natural Cesarean delivery	95 (67.4) 46 (32.6)
Breastfeeding	Never Till 3 months Till 6 months Till 1 year More than 1 year	7 (5.0) 31 (22.0) 19 (13.5) 51 (36.2) 33 (23.4)
Formula feeding	Never Till one year Till 1.5 years More than 1.5 years	53 (37.6) 24 (17.0) 44 (31.2) 20 (14.2)
Age of starting to feed with spoon	Younger than 6 months 6–9 months 9–12 months 1–3 years	1 (0.7) 104 (73.8) 27 (19.1) 9 (6.4)
Start of kindergarten attendance	Younger than 1 year old 1–3 years More than 3 years	3 (2.1) 116 (82.3) 21 (14.9)
Oral health-related behaviors		
Nail biting	Yes	28 (19.9)
Sleeping with open mouth	Yes	22 (15.6)
Bruxism during night	Yes	23 (16.3)
Snoring at night	Yes	12 (8.5)
Thumb sucking	Has never sucked Till 1 year 1–3 years Still is sucking	64 (45.4) 61 (43.3) 5 (3.5) 11 (7.8)
Grazing vegetables	Never Once a month or less Several times a week Every day	2 (1.4) 5 (3.5) 82 (58.2) 52 (36.9)
Drinking carbonated drinks	Never 2–3 times/month Every day	69 (48.9) 70 (49.6) 2 (1.4)
Eating sweets	Several times a month Once a month Several times a day Every time a child wants it	28 (19.9) 71 (50.4) 23 (16.3) 19 (13.5)
Times teeth cleaned	Only by parents’ reminder Once a day Twice a day	10 (7.1) 45 (31.9) 86 (61.0)
Cleaning teeth	Child by themself Parents Child by themself with parents’ help	69 (48.9) 5 (3.5) 67 (47.5)
Age when cleaning teeth started	With the first tooth After appearance of anterior teeth At the age of 1 year	79 (56.0) 22 (15.6) 40 (28.4)
Last visit to a dentist	Never More than 2 years ago 2 years ago 1 year ago Less than 6 months ago	11 (7.8) 1 (0.7) 4 (2.8) 34 (24.1) 91 (64.5)
Main source of information about child dental care *		
From relatives	Yes	2 (1.4)
From friends	Yes	61(43.3)
From general physician	Yes	89 (63.1)
From internet	Yes	79 (56.0)
From books/magazines	Yes	81 (57.4)
Satisfaction with information received from general physician	No information received Expected more information Information was satisfactory Information was useful	18 (12.8) 15 (10.6) 70 (49.6) 38 (27.0)

* Each mother had the possibility to mention several sources of information; therefore, the total percent for all these answers is higher than 100%.

**Table 2:** Oral health-related behaviors and information that mothers received from friends.

Variable, *N* = 141	Group	Information from Friends, N (%)		*p* Value
		Not Received, N = 80 (55.7%)	Received, N = 61 (44.3%)	
Thumb sucking	Has never sucked Till 1 year More than 1 year	41 (64.1) 28 (45.9) 11 (68.8)	23 (35.9) 33 (54.1) 5 (31.3)	0.07
Grazing vegetables	Never/once a month or less Several times a week Every day	28 (53.8) 46 (56.1) 6 (85.7)	24 (46.2) 36 (43.9) 1 (14.3)	0.27
Drinking carbonated drinks	Never 2–3 times/month	47 (68.1) 31 (44.3)	22 (31.9) 39 (55.7)	< 0.01
Eating sweets	Several times a month Once a month Several times a day Every time a child wants it	19 (67.9) 38 (53.5) 13 (56.5) 10 (52.6)	9 (32.1) 33 (46.5) 10 (43.5) 9 (7.4)	0.60
Times teeth cleaned	Only by parents’ reminder/ once a day Twice a day	32 (58.2) 48 (55.8)	23 (41.8) 38 (44.2)	0.46
Cleaning teeth	Child by themself Parents or with their help	45 (65.2) 35 (48.6)	24 (34.8) 37 (51.4)	0.03
Age when cleaning teeth started	With the first tooth After appearance of anterior teeth At the age of 1 year	37 (46.8) 16 (72.7) 27 (67.5)	42 (53.2) 6 (27.3) 13 (32.5)	0.03
Last visit to a dentist	1 year ago Less than 6 months ago 2 years ago, or more	49 (53.8) 17 (50.0) 14 (87.5)	42 (46.2) 17 (50.0) 2 (12.5)	0.03

**Table 3:** Diseases that affect oral health and results of physical examination related to tongue thrust.

Variable, *N* = 141	Group	Tongue Thrust, *N* (%)		*p* Value
		Normal, *N* = 66 (46.8%)	Infantile, *N* = 75 (53.2%)	
Diseases that affect oral health				
Allergies	No Yes	50 (45.0) 16 (53.3)	61 (55.0) 14 (46.7)	0.27
Long-lasting flu	No Yes	39 (51.3) 27 (41.5)	37 (48.7) 38 (58.5)	0.16
Face/head/neck traumas	No Yes	54 (46.2) 12 (50.0)	63 (53.8) 12 (50.0)	0.45
Surgery under general anaesthesia	No Yes	55 (45.1) 11 (57.9)	67 (54.9) 8 (42.1)	0.21
Otitis media	No Yes	57 (51.8) 9 (29.0)	53 (48.2) 22 (71.0)	0.02
Results of physical examination				
Lip power (g), mean (SD)		368.3 (134.4)	338.8 (134.3)	0.13
Breathing, N (%)	Nose Mouth	61 (53.0) 5 (19.2)	54 (47.0) 21 (80.8)	<0.01
Speech, N (%)	Normal Non-normal	51 (58.0) 15 (28.3)	37 (42.0) 38 (71.7)	<0.01
Interdental speech, N (%)	Normal Lisping	65 (52.4) 1 (5.9)	59 (47.6) 16 (94.1)	<0.01
Occlusion, N (%)	Normal Other than normal	27 (54.0) 39 (42.9)	23 (46.0) 52 (57.1)	0.14

**Table 4:** Association between children’s orofacial health and tongue thrust.

Variable	B	S.E.	Odds ratio, OR	95% Confidence interval, CI	*p* Value
Breath	1.53	0.56	4.60	1.55; 13.65	0.01
Speech	1.23	0.40	3.43	1.58; 7.48	<0.01
Otitis media	0.96	0.47	2.60	1.04; 6.50	0.04
Gender	0.80	0.38	1.08	0.52; 2.27	0.83
Age	−0.09	0.36	0.92	0.45; 1.87	0.82

## Data Availability

The data presented in this study are available on request from the corresponding author. The data are not publicly available due to its sensitive nature.
